# Cultural adaptation of a pediatric functional assessment for rehabilitation outcomes research

**DOI:** 10.1186/s12913-017-2592-6

**Published:** 2017-09-15

**Authors:** Kristen E. Arestad, David MacPhee, Chun Y. Lim, Mary A. Khetani

**Affiliations:** 1 Department of Occupational Therapy, Colorado State University, Occupational Therapy Building, Fort Collins, CO 80523-1573 USA; 2 Department of Human Development and Family Studies, Colorado State University, Behavioral Sciences Building 319, Fort Collins, CO 80523-1570 USA; 3 Department of Child Development, KK Women’s and Children’s Hospital, Rd, #05-06 Boon Siew Building, 75 Bukit Timah, 229833 Singapore; 4 Department of Occupational Therapy, University of Illinois at Chicago, 1919 West Taylor Street, Room 316A, Chicago, IL 60612-7250 USA

**Keywords:** Culture, Translation, Adaptation, Feasibility, Assessment, Questionnaire

## Abstract

**Background:**

Significant racial and ethnic health care disparities experienced by Hispanic children with special health care needs (CSHCN) create barriers to enacting culturally competent rehabilitation services. One way to minimize the impact of disparities in rehabilitation is to equip practitioners with culturally relevant functional assessments to accurately determine service needs. Current approaches to culturally adapting assessments have three major limitations: use of inconsistent translation processes; current processes assess for some, but not all, elements of cultural equivalence; and limited evidence to guide decision making about whether to undertake cultural adaptation with and without language translation. The aims of this observational study are (a) to examine similarities and differences of culturally adapting a pediatric functional assessment with and without language translation, and (b) to examine the feasibility of cultural adaptation processes.

**Methods:**

The Young Children’s Participation and Environment Measure (YC-PEM), a pediatric functional assessment, underwent cultural adaptation (i.e., language translation and cognitive testing) to establish Spanish and English pilot versions for use by caregivers of young CSHCN of Mexican descent. Following language translation to develop a Spanish YC-PEM pilot version, 7 caregivers (4 Spanish-speaking; 3 English-speaking) completed cognitive testing to inform decisions regarding content revisions to English and Spanish YC-PEM versions. Participant responses were content coded to established cultural equivalencies. Coded data were summed to draw comparisons on the number of revisions needed to achieve cultural equivalence between the two versions. Feasibility was assessed according to process data and data quality.

**Results:**

Results suggest more revisions are required to achieve cultural equivalence for the translated (Spanish) version of the YC-PEM. However, issues around how the participation outcome is conceptualized were identified in both versions. Feasibility results indicate that language translation processes require high resource investment, but may increase translation quality. However, use of questionnaires versus interview methods for cognitive testing may have limited data saturation.

**Conclusions:**

Results lend preliminary support to the need for and feasibility of cultural adaptation with and without language translation. Results inform decisions surrounding cultural adaptations with and without language translation and thereby enhance cultural competence and quality assessment of healthcare need within pediatric rehabilitation.

## Background

Significant racial and ethnic health care disparities, such as those experienced by Hispanic children with special health care needs (CSHCN), indicate gaps in enacting culturally competent health care, including within pediatric rehabilitation. Cultural competence is the ability to effectively and appropriately interact cross-culturally [1]. Health disparities for Hispanic CSHCN are often described in three ways: (a) access to services, (b) quality care, and (c) service-related outcomes. This study focuses on culturally informed assessment of health care needs, which is essential to reducing each type of health disparity.

Racial and ethnic disparities in service access have been identified for Hispanic children according to their insurance coverage [2], health screenings [3], and community-based services (e.g., early intervention, pediatric rehabilitation). For CSHCN who have access to a primary care provider, nearly 22.5% of Hispanic CSHCN report having ‘moderate or big’ problems obtaining special health care services as compared to 13% of non-Hispanic White children. Further, non-White families, including Hispanic families, have more difficulty obtaining referrals when in need of specialty care [4] and are less likely than non-Hispanic White children to have a usual or specific source of ongoing health care [5].

Among those who obtain access, disparities exist with regards to quality care and service-related outcomes. Caregivers of Hispanic CSHCN appear to be less satisfied with their children’s services when compared to other major racial and ethnic groups [3], as evidenced by poor provider-patient communication, care coordination, and family-centered care [5]. In comparison to other major racial and ethnic groups, caregivers of Hispanic CSHCN also report higher rates of unmet therapy needs (i.e., physical therapy, occupational therapy, speech therapy) [3]. Further, race and ethnicity are strong predictors of health status outcomes (e.g., medical status) for children participating in Part C early intervention (EI) where non-Hispanic White children are healthier than minority children [6]. Moreover, health disparities between White and non-White children become larger between EI entry and exit at 36 months after controlling for health status at EI entry [6]. Additionally, families of minority children are approximately twice as likely to have less positive family outcomes (e.g., perceived family quality of life) upon their discharge from EI than families of White children [6]. Similarly, Hispanic preschoolers who had received EI services were more likely to experience community participation difficulties, when compared to non-Hispanic White preschoolers [7].

One way to minimize health care disparities is to ensure that practitioners can enact culturally competent care during assessment, intervention planning, and intervention. As the first of these tasks, culturally competent assessment practices are especially critical to initial provider-client interaction and care plan development. Practitioners need to be able to effectively and efficiently conduct quality baseline assessment using culturally valid and reliable approaches to assessment [8]. Due to time and costs associated with measure development, it is often more feasible to culturally adapt and validate an assessment from one culture for use in a different culture than to create new measures for specific cultural groups [9].

Cultural adaptation involves establishing the equivalency and relevancy of an assessment from its source language and culture to the target population [9–11]. Most efforts to increase the accessibility of measures for use with diverse clients typically emphasize language translation [9, 12]. However, language translation alone is insufficient, because it captures only the etic perspective; however, further work is needed in order to capture the emic perspective to account for cultural differences that might alter the content of an assessment (i.e., instructions, questions, examples, scales) and how the assessment is administered (e.g., questionnaire, interview) [9, 12, 13]. To our knowledge, there is no gold standard cultural adaptation framework, and a comparison of existing frameworks reveals several limitations: (a) process guidelines lack agreement; (b) discrepancies exist in what is required to achieve cultural equivalence; and (c) no known frameworks address cultural adaptation without the need for translation. The present study aims to address each of these limitations.

Cultural adaptation commonly involves forward and back translation and committee review [9, 10, 14, 15]. Discrepancies exist around (a) the number of translators used and their qualifications, (b) whether to perform back translation processes on each forward translation or a synthesized version, (c) recruiting additional personnel for synthesis processes needed to produce a single translated version, (d) committee review composition and size, and (e) sample size recommendations for pilot testing [9, 10, 14, 15]. These discrepancies may be related to feasibility, such as the lack of available qualified translators and cost.

Current frameworks and culturally adapted assessments have proposed different equivalence requirements and utilize different terms to assess for cultural equivalency. Cultural equivalency is “the extent to which an instrument is equally suitable for use in two or more cultures” [11], (p1257). To achieve cultural equivalency, frameworks emphasize assessing for semantic, idiomatic, item (also called experiential and content), conceptual, measurement, and operational equivalencies (also called technical) [9–11]. As shown in Table 1, semantic, item, and conceptual equivalencies are most commonly emphasized in current frameworks.

**Table 1:** Cultural equivalency dimensions across cultural adaptation frameworks

Cultural adaptation frameworks	Equivalence type					
	Semantic	Idiomatic	Item	Conceptual	Measurement	Operational
Guillemin et al., [9]	✓	✓	✓	✓		
Beaton et al., [10]	✓	✓	✓	✓		
Sousa & Rojjanasriat, [14]	✓		✓	✓	✓	
Stevelink & van Brakel, [11]	✓		✓	✓	✓	✓

*Semantic equivalence* is the degree to which word meanings transfer across languages and cultures. *Idiomatic equivalence* is the degree to which idiomatic and colloquial expressions translate across languages and cultures. *Item equivalence* is the degree to which item traits or experiences are relevant and acceptable across cultures. *Conceptual equivalence* is the degree to which concepts within assessment items have the same meaning across cultures. *Measurement equivalence* is the degree to which the psychometric properties of the original and adapted versions are equivalent*. Operational equivalence* is the degree to which the measurement methods (e.g., format, mode of administration) are appropriate across cultures

Similarly, semantic, item, and conceptual equivalencies are most commonly assessed in culturally adapted pediatric assessments with outcomes relevant to pediatric rehabilitation (i.e., quality of life, participation, performance, functional skills) as evaluated through reported assessment of equivalency types as well as comparison of equivalency definitions and the reported processes used (see Table 2) [16–22]. Pediatric assessments commonly address semantic equivalence during translation. However, while these adapted children’s assessments emphasize item and conceptual equivalencies, there is less clarity about the methods used to achieve item and conceptual equivalencies. Only the cultural adaptation of PEDI for use with children in Puerto Rico outlined methods for assessing item and conceptual equivalencies; however, limitations exist in the methods employed [19]. Limitations include: (a) suggestions for changes and additional feedback were not elicited from experts included in the study; and (b) no feedback was elicited from caregivers or children.

**Table 2:** Cultural equivalency dimensions addressed in culturally adapted assessments of children

Assessment	Equivalence Type					
	Semantic	Idiomatic	Item	Conceptual	Measurement	Operational
Youth Quality of Life Instrument - Research Version (YQOL-R): Spanish version [16]	✓		✓			✓
Children’s Assessment of Participation and Enjoyment and Preferences for Activities of Children (CAPE/PAC): Spanish version [17]	✓		✓	✓	✓	
Perceived Efficacy and Goal Setting System (PEGS): German version [18]	✓	✓	✓	✓		
Pediatric Evaluation of Disability Inventory (PEDI): Puerto Rican version [19]	✓		✓	✓	✓	✓
Preschool Activity Card Sort (PACS): Spanish version [20]	✓					
Paediatric Asthma Quality of Life Questionnaire (PAQLQ): Spain version [21]	✓		✓	✓	✓	
CAPE/PAC: Swedish version [22]	✓		✓	✓	✓	

Due to increasing prevalence of multiple cultural groups sharing a common language (e.g., Spanish-speaking clients may represent a wide range of cultural groups, including individuals from Mexico, Spain, and Venezuela), the development of a cultural adaptation framework that does not require translation is needed to facilitate culturally competent assessment practices. In the absence of such a framework, the Young Children’s Participation and Environment Measure (YC-PEM) is a pediatric functional assessment that was culturally adapted for use in Singapore, a primarily English-speaking country [23]. Semi-structured interviews with providers and cognitive interviews with caregivers were used to inform content revisions related to item, semantic, and operational equivalencies.

The most common approaches to culturally adapting an assessment in preparation for psychometric testing involve assessing for (a) semantic and idiomatic as well as (b) item and conceptual equivalencies. Semantic equivalence is examined during the translation process by assessing the transfer of word meanings across cultures. Although idiomatic equivalence appears to be less commonly addressed, it likely gets assessed within semantic equivalence as both equivalencies address the transferability of words and phrases across cultures. Item and conceptual equivalencies are typically evaluated in terms of how relevant and appropriate the items and assessment concepts are to the end-user. Although prior literature lacks clarity on how to carry out these processes, they often involve focus groups, expert panels, or smaller samples of individuals from the target population and employ cognitive testing methodology [16–19, 22, 24]. Lack of clarity in how to address item and conceptual equivalencies poses two potential problems: (a) it potentially limits the trustworthiness of existing culturally adapted measures; and (b) it limits the advancement of cultural adaptation processes, thereby limiting the development of high quality culturally adapted assessments. Although utilized in cultural adaptations, operational equivalence is less frequently addressed; however, it typically involves identifying accessible formats (e.g., interview, paper, or online questionnaire) based on participant characteristics [11].

This study seeks to address these limitations in the context of culturally adapting the YC-PEM [25], a validated patient-reported functional outcome measure, in English (no translation) and Spanish (translation) for potential use by caregivers of Hispanic CSHCN ages 0 to 5 years old living in the United States. To our knowledge, this is the first study to systematically develop and compare multiple versions of a culturally adapted questionnaire for potential use by a Hispanic population of young CSHCN.

The purpose of this study is to (a) compare the English and Spanish pilot YC-PEM versions to identify similarities and differences of culturally adapting an instrument with and without translation, and (b) examine the feasibility of developing culturally adapted English and Spanish versions of the YC-PEM for potential use by a Hispanic population of CSHCN. We had three specific aims:

1. To identify revisions required to achieve semantic and idiomatic equivalencies when developing culturally adapted versions of the YC-PEM. We hypothesize that a greater number of changes are required to achieve semantic and idiomatic equivalencies of the culturally adapted and translated version of the YC-PEM than the non-translated version.
2. To identify revisions required to achieve item and conceptual equivalencies when developing culturally adapted versions of the YC-PEM. We expect a similar number of changes required to achieve item and conceptual equivalencies for the English and Spanish YC-PEM versions.
3. To examine the feasibility of developing culturally adapted English and Spanish pilot versions of the YC-PEM.

Study results will provide evidence to inform continued use of contemporary guidelines for culturally adapting patient-reported functional outcome measures with and without language translation.

## Methods

### Study design

This cross-sectional study employed quantitative and qualitative methods framed within a cognitive testing approach. In creating questionnaires, cognitive testing is used to identify problems associated with interpreting and measuring the intended construct [24]. Cognitive testing typically entails administering a preliminary version to individuals from the target population (i.e., the end-user of the instrument) followed by eliciting information about the questionnaire items and the users’ chosen responses. This approach has been used to culturally adapt measures, including assessment of item relevance [16, 17] and comprehensiveness of item set to the culture [19], as well as generation of activities relevant to the culture [18, 22]. Thus, cognitive testing data were used to inform required revisions to achieve cultural equivalency of the English and Spanish YC-PEM versions.

### Participants

Participants were recruited through convenience sampling by early intervention providers and the primary investigator as part of a larger study underway *(N =* 37). The primary early intervention programs are major service providers in the Denver Metro catchment area. There is considerable variability in sample size recommendations for pilot testing culturally adapted assessments, ranging from 5 to 40 participants [10, 14, 15]. For this study, eight caregivers of young children ages 0 to 5 years old—five with Spanish as their primary language; three with English as their primary language—enrolled in this study.

The respondents met the following inclusion criteria: (a) self-identified as a parent or legal guardian who is 18 years or older; (b) self-identified as a caregiver of a child of Mexican descent between the ages of 0 and 5; (c) resided in the United States; and (d) were able to read and write in English or Spanish. Although the Hispanic population includes a wide variety of cultures, recruitment occurred from within a population of Mexican descent, because it is the largest cultural group within the United States and Colorado, where the majority of participants were recruited [26]. One Spanish-speaking participant was excluded due to limited literacy level and item responses indicating lack of comprehension.

### Measures

#### Acculturation rating scale for Mexican Americans-II (ARSMA-II)

The ARSMA-II [27] is a self-report questionnaire that was administered to assess for caregiver acculturation status. Acculturation status refers to the extent to which the cultural patterns of individuals change along a continuum when they live in a culture different from their cultural origin. Thus, acculturation was assessed to determine if variations in acculturation status were related to revisions needed for cultural adaptations with and without translation. This study utilized one of two ARSMA-II scales assessing for separation and marginalization dimensions of acculturation. This scale has 30 items rated on a 5-point scale, from 1 (*not at all*) to 5 (*extremely often or almost always*). The first 17 items are summed to create a Mexican Orientation Score (MOS) and the remaining 13 items comprise an Anglo Orientation Score (AOS). ARSMA-II uses a bilingual format with both English and Spanish versions displayed side by side. The mean MOS subscale is then subtracted from the mean AOS subscale, resulting in an acculturation score from very Mexican oriented (< −1.33) to very Anglo oriented (> 2.45).

ARSMA-II has shown good internal consistency for the AOS (α = .83) and MOS (α = .88) subscales [27]. Test-retest reliability is excellent for AOS (ICC = .94) and MOS (ICC = .96) subscales.

#### Young Children’s participation and environment measure (YC-PEM)

The YC-PEM [25] has undergone validation for use by caregivers of young children between 0 and 5 years old with and without disabilities and delays [28–30]. The YC-PEM evaluates caregivers’ perceptions of their young child’s participation in activities and environmental impact on participation.

The YC-PEM assesses young children’s participation in broad activity types across three settings: home, school, and community. For each activity, the parent is asked to report on frequency, involvement level, and their desire for change in their child’s participation. If the caregiver reports “yes” for change desired, the caregiver is asked to specify the type of change desired and up to three strategies that they have used to promote their child’s participation in activities of that type. Caregivers then report on the impact (i.e., supports and barriers) of the environment on their child’s participation for each setting. The environmental sections conclude by asking caregivers to describe up to three strategies they utilize to promote their child’s participation in that setting.

Across measurement dimensions, the YC-PEM has shown fair to excellent internal consistency for the home (α = .82 to .96), daycare/preschool (α = .67 to .92), and community (α = .68 to .96) [28]. Additionally, the YC-PEM has shown poor to excellent test-retest reliability for the home (ICC = .57 to .91), daycare/preschool (ICC = .31 to.92), and community (ICC = .52 to .94) settings. The YC-PEM was recently endorsed as a supplemental outcome measure to capture common data elements related to engagement and quality of life in studies involving children with cerebral palsy and other neurological disorders [31]. To our knowledge, the first culturally adapted YC-PEM was recently pursued for use in Singapore, which has been reported as retaining many similar psychometric properties [32].

### Data collection

Institutional Review Board approval was obtained prior to recruitment and the two-phase data collection. Phase 1 (May 2015–August 2015) involved translation of the YC-PEM to create a Spanish version. Phase 2 (October 2015–February 2016) involved the pilot and cognitive testing of the original English and translated Spanish versions of the YC-PEM for use with caregivers of CSHCN of Mexican descent who were between 0 and 5 years old. For Phase 2, participants completed a consent form, demographic questionnaire, and the ARSMA-II Scale 1 in their preferred language (English or Spanish) prior to completing the English or Spanish version of the YC-PEM with accompanying cognitive testing questions. Each participant received a $50 payment.

#### Phase 1: Spanish translation

Translation procedures and sample size were based on best practice guidelines for measurement translation. Forward translations (FT1, FT2, and FT3) were produced by three qualified translators who were bilingual with Spanish as their first language. One of the forward translators was familiar with the YC-PEM, and the other two forward translators were unfamiliar with the instrument. The synthesized forward translation (FT123) was produced by having research staff compare FT1, FT2, and FT3 with the original YC-PEM and working with the forward translators to achieve consensus on the FT123 version via email discussion.

Back translations (BT1 and BT2) were produced from the FT123 version by two qualified translators who are bilingual with English as their first language. One of the back translators was familiar with the YC-PEM and the other back translator was unfamiliar with the instrument. The synthesized back translation (BT12) was produced by having study staff compare BT1 and BT2 with the original YC-PEM and working together with the two back translators to achieve consensus on the BT12 version.

An expert committee was assembled to conduct the final review of all translations (i.e., the original, FT1, FT2, FT3, FT123, BT1, BT2, BT12) in order to resolve discrepancies and create a Spanish pilot version. The committee consisted of the three forward translators, two back translators, a member of the original instrument development team, and an additional bilingual member of the research team. Each committee member independently reviewed the produced translations and original YC-PEM. When committee members identified a discrepancy, they were prompted to provide a suggestion for alternate wording to resolve the discrepancy. Discrepancies were not considered resolved until committee consensus was reached. Each translator was compensated for his or her time.

#### Phase 2: Cognitive testing

Early intervention providers guided eligible and interested families to consent online or via paper forms when computer and internet access were not available. Participants were then administered study questionnaires via email (for electronic versions; *n* = 3, all in English) or service provider (for paper versions; *n* = 5, all in Spanish). For cognitive testing, a written probing technique was integrated into the YC-PEM questionnaire to allow for more feasible data collection as compared to interview methods. Response formats included closed-ended and open-ended questions to gather feedback on suggested changes.

### Data analysis

#### Preliminary analyses—Missing data

For the ARSMA-II, one case contained one missing value; however, when substituting the missing value with either the minimum or maximum scale values, no change occurred to the acculturation level. Thus, missing data did not significantly affect acculturation levels.

For the cognitive testing items, identification of missing data is not possible because many items do not require a response unless the participant deems that revisions are necessary. Therefore, to approximate missing data from the cognitive testing questions, YC-PEM completion rates were examined. For the YC-PEM home section, mean completion rates were 67.0% for the participation section (range: 5.1–100) and 84.6% for the environment section (range: 0–100). One participant had missing data across all 13 home environmental items. For the community section, mean completion rates were 72.7% for the activity section (range: 6.1–100) and 84.0% for the environment section (range: 0–100). One participant had missing data across all 17 community environmental items.

There were missing data in six out of seven cases, resulting in 186 instances of missing data. In 55.4% of these instances, participants stated that their child was too young. Potential formatting concerns when completing the YC-PEM in PDF or paper formats accounted for 24 of the missing values (12.4%) as identified by consistent errors in item completion. Finally, one participant provided no responses for all “desire change” items and left two pages blank resulting in 45 missing values (24.2%).

#### Main analyses

Qualitative data gathered from cognitive testing were first coded to three cultural equivalence types: (a) semantic/idiomatic, (b) item, and (c) conceptual. The primary investigator and two additional research staff initially coded data. Inter-rater agreement was 64.8% after initial coding. Discrepancies were resolved by consensus among coders through phone discussion after which inter-rater agreement was 98.9%. A fourth member of the research team then coded all discrepancies to ensure dependability of study findings due to the high initial discrepancy rate, resulting in 100% inter-rater agreement. Coded data were then analyzed to address the first two study aims as described below. Principles of data saturation were considered for the analysis and interpretation of qualitative data. Data saturation occurs when new information is no longer elicited from participants [33].

##### Aim 1: Semantic and idiomatic equivalencies.

Frequency counts were used to describe changes required for achieving semantic and idiomatic equivalencies in each culturally adapted YC-PEM version (English or Spanish). Changes in wording that differed from direct translation of the original version (Spanish version) or original wording (English version) were counted. For the Spanish version, a total count was calculated by summing together the total number of semantic and idiomatic changes that occurred during the translation process (Phase 1) as well as coded responses to cognitive testing items. For the English version, a total count was calculated by summing coded participant responses to cognitive testing questions.

##### Aim 2: Item and conceptual equivalencies

Frequency counts were used to identify the number of proposed revisions required to establish item and conceptual equivalencies in each YC-PEM pilot version in order to examine the effect of language on the revisions required.

To establish trustworthiness, multiple coders and self-reflexivity were used to ensure authenticity of findings [33]. Four separate coders with different levels of knowledge on the topic of cultural adaptation were recruited. All coders were current or former members of the same research team and had worked consistently with the YC-PEM, but had varying disciplinary backgrounds (i.e., rehabilitation science, psychology, applied developmental science) and professional backgrounds (i.e., occupational therapy, early childhood education), which resulted in varying levels of knowledge about cultural adaptation and functional assessments.

Self-reflexivity is used to acknowledge the experiences that a researcher brings to data collection and/or analysis that can impact the findings in order to establish trustworthiness. The primary investigator has Argentine cultural heritage, thus potentially influencing interpretations about the influences of Mexican-American culture with regards to cultural differences in children’s participation, barriers and supports to participation, and strategies to promote participation-level outcomes. The primary investigator also has prior professional experience working with Mexican-American students receiving special education services. In this position, she noticed discrepancies in the cultural appropriateness of interventions to improve educational outcomes for these students in comparison with their European-American peers, which has since influenced her focus on cultural competence. Thus, these experiences helped shape study hypotheses as she has anecdotally observed the influences of culture on participation.

##### Aim 3: Feasibility of cultural adaptation

To examine feasibility of cultural adaptation, language translation and cognitive testing processes were examined with respect to resources required, data collection procedures, and data quality [34, 35]. Completion time was tracked for each phase of translation. To determine the quality of translation data, frequency counts of discrepancies among translators were done for each phase of the translation. Discrepancies were counted at the item/sentence level. For cognitive testing, resources examined included time for data collection, available methods of questionnaire administration, and the role of early intervention providers. Recruitment rates were examined to identify barriers to recruitment. Data collection procedures were examined to identify suitability of the procedures for the targeted population and to examine cultural adaptation outcomes.

## Results

### Participant characteristics

Participants included seven caregivers (four Spanish-speaking; three English-speaking) of CSHCN of Mexican descent ages 0 to 5 years old. All children reported on were either developmentally delayed or at-risk for developmental delay. All participants self-identified as mothers or female legal guardians of the child reported on. Most participants reported having no paid employment (85.7%) and having a child between the ages of 1 and 36 months old. Education levels were higher for the English-speaking group with 66.7% having received an Associate’s degree, whereas the highest level of education reported for the Spanish-speaking group was some college or technical training (50.0%). Household income levels were higher for English-speaking participants with 66.7% reportedly earning $60,000 or more annually, whereas 75% of Spanish-speaking participants reported an annual income of $60,000 or less.

In terms of acculturation, all Spanish-speaking participants identified at Level 1 (i.e., very Mexican oriented) and 2 (Mexican oriented to approximately balanced bicultural) acculturation levels. In contrast, all English-speaking participants identified at Level 4 (i.e., strongly Anglo oriented) and 5 (i.e., very assimilated) acculturation levels.

### Aim 1: Semantic and idiomatic equivalencies

As shown in Table 3, the Spanish version of the YC-PEM required a greater number of revisions than the English version to achieve semantic and idiomatic equivalencies. Most revisions for the Spanish version were identified during language translation (87.0%); however, 13.0% of the revisions were identified through cognitive testing. Table 4 provides examples of the revisions required to achieve semantic and idiomatic equivalencies for the Spanish version.

**Table 3:** Proposed Revisions for Semantic/Idiomatic Equivalence

Pilot Version	YC-PEM				
	Instructions	Home	Daycare/Preschool	Community	Total
Spanish					
Total (*n)*	22	52	20	29	123
Translation (%)	95.4	75.0	100.0	93.1	87.0
Cognitive testing (%)	4.5	25.0	–	6.9	13.0
English					
Cognitive testing (*n*)	–	1	–	–	1

**Table 4:** Sample revisions proposed for semantic/idiomatic equivalence of Spanish version

Original	Modification *(English translation)*
Workbooks	Libros de juegos *(Game books)*
Having ramps	Tener rampas para sillas de ruedas *(Having ramps for wheelchairs)*
Policies	Reglas/reglamentos *(Rules/regulations)*
Keeping current	Mantenerse informada *(Staying informed)*

### Aim 2: Item and conceptual equivalencies

The Spanish version of the YC-PEM required a greater number and range of revisions as compared to the English version for item and conceptual equivalencies (see Table 5). For item equivalence, participants who completed the Spanish version suggested revisions for home and community sections of the YC-PEM. Table 6 provides examples of proposed revisions to achieve item equivalence, most of which pertained to examples of activities and environmental factors and resources.

**Table 5:** Proposed revisions for item and conceptual equivalencies

Pilot version	Item equivalence	Conceptual equivalence
Spanish		
Total (*n*)	24	23
Instructions(%)	–	21.7
Home (%)	66.7	65.2
Community (%)	33.3	13.0
English		
Total (*n*)	1	3
Instructions (%)	–	33.3
Home (%)	–	66.7
Community (%)	100.0	–

**Table 6:** Sample revisions proposed for item equivalence for Spanish version

Administered item		Proposed modification
Spanish	*English translation*	Spanish *(English)*
Celebraciones en el hogar (ej.: reuniones de días festivos, fiestas de cumpleaños)	*Celebrations at home (*e.g.*, holiday gatherings, birthday parties)*	Modify: Change “Celebraciones en el hogar” (*Celebrations at home)* to “Reuniones de hogar” *(Home gatherings)*
Reuniones y actividades religiosas o espirituales (ej.: asistir a lugares de adoración, clases y grupos religiosos)	*Religious or spiritual gatherings and activities (*e.g.*, attending places of worship, religious classes and groups)*	Add examples: Misas *(Masses)*; Pastorelas *(Nativity plays)*; Catecismo *(Catechism)*
Actividades físicas no estructuradas (ej.: áreas de juego y parques, playas, caminatas, andar en bicicletas y patinetas, andar en trineos, ir de pesca, patinar en el hielo)	*Unstructured physical activities (*e.g.*, playgrounds and parks, beaches, hiking, bikes and scooters, sledding, fishing, ice skating)*	Add examples: Albercas *(Pools)*; Carreras en el campo al aire libre *(Races outside)*; Brincar la cuerda *(Jump rope)*

Responses from six participants (four Spanish-speaking; two English-speaking) indicated some limitations in understanding the concept of participation (i.e., conceptual equivalence). Table 7 provides examples of participant responses that indicated some discrepancies between how they conceptualized participation in comparison to the description of the concept in the YC-PEM survey instructions. Common issues that arose related to these discrepancies included age, disability status, and independence.

**Table 7:** Sample participant feedback related to conceptual equivalence

YC-PEM Concepts	Participant Comments
Participation	“N/A - does not apply to the age of my son” “She doesn’t do any activity because she is a baby.” *(Ella todavía no hace ninguna actividad porque es una bebe.)* “He is too young to do his self care.” *(Es pequeño para hacer su mantenimiento.)* “It is very difficult [to select responses] because my daughter can’t fend for herself because she has an intellectual delay.” *(Es muy difícil porque mi hija no puede valerse por si sola tiene un retraso en su cerebro.)*
**Participation Dimensions**	
Involvement	**Define “involved”** “It is the opportunity provided for their physical and mental development. Through activities, techniques, and strategies, the child can develop, improve, or acquire skills and abilities.” *(Es la oportunidad que se le brinda para su desarrollo físico y mental. A través de actividades, técnicas y estrategias, el niño puede desarrollar, incrementar ó adquirir habilidades y destrezas.)* “What he does alone and with help.” *(Que es lo que el hace solo y con ayuda.)* “For a child to be involved in an activity, … they also need to be able to do things on their own.” **Describe how you selected “how involved” your child is in an activity** “Response [for Celebrations at Home] is based on how often my child has a sensory meltdown and refuses to participate during these celebrations.” “She is not involved because she doesn’t know how to do it herself if alone.” (*No está involucrado porque no sabe valerse por si sola.*)
Frequency	“Response is based on how often my child is combative during the bedtime routine.” “My daughter cannot participate because of her condition.” (*Mi hija no puede participar por su condición.*)

### Aim 3: Feasibility of cultural adaptation (language translation and cognitive testing)

#### Language translation

**Fig. 1:**
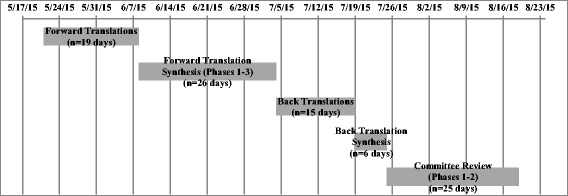
Spanish translation process timeline

The Spanish translation of the YC-PEM was pursued in five phases and took 91 days to complete (see Fig. 1 for process timeline). More than half (56%) of the time was dedicated to the forward translation synthesis and committee review.

**Fig. 2:**
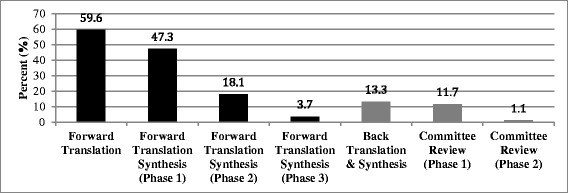
Percent discrepancies during Spanish translation of pediatric functional assessment

Quality of the translation was examined according to discrepancies among translators for each translation phase and trends in the number of discrepancies reported over time (see Fig. 2 for discrepancies throughout the translation process). There were discrepancies in 141 out of 188 items (75.0%) as detected during forward translation (FT1, FT2, FT3); however, only 112 items (59.6%) had discrepancies that were not redundant to previously identified discrepancies (e.g., same word discrepancy). Discrepancies reduced throughout the forward translation synthesis process with distinct discrepancies present in 89 items (47.3%) in the first phase, 34 items (18.1%) in the second phase, and 7 items (3.7%) in the third phase. Discrepancies increased to 25 items (13.3%) with the back translation and synthesis phase. Discrepancies again reduced throughout the committee review process with discrepancies present in 22 items (11.7%) in the first phase and 20 items (10.6%) in the second phase. However, only 2 items (1.1%) in the second committee review phase contained discrepancies related to equivalency concerns while the remaining identified discrepancies concerned spelling/grammatical errors (*n* = 6; 3.2%) and committee member wording preference (*n* = 12; 6.4%).

#### Cognitive testing

A total of 8 of the 23 (34.8%) eligible families enrolled during a 16-week period. Participants declined due to lack of interest (*n* = 3), too busy/stressed (*n* = 8), privacy concerns (*n* = 1), lost completed materials (*n* = 1), or were lost to follow up (*n* = 2). Most eligible caregivers did not have regular computer or internet access (*n* = 16).

## Discussion

Existing racial and ethnic heath care disparities illuminate gaps in enacting culturally competent care. To enhance cultural competence within assessment practices, cultural adaptation can be used to ensure that assessments adequately capture outcomes for clients across cultures.

To our knowledge, this is the first study to examine similarities and differences in culturally adapting an instrument with and without language translation. Additionally, this study generates new knowledge surrounding the feasibility of carrying out cultural adaptation processes (i.e., language translation and cognitive testing). Study findings provide preliminary evidence to help guide decision making regarding cultural adaptation processes and the relative costs and benefits of cultural adaptation with and without language translation. Throughout the remainder of this section, each set of study findings is discussed in detail.

### Semantic and idiomatic equivalencies

Results of this study support the hypothesis that more revisions are required to achieve semantic and idiomatic equivalencies of the YC-PEM Spanish version as compared to the YC-PEM English pilot version. These findings are congruent with the emphasis on using language translation to achieve sematic and idiomatic equivalence in cultural adaptation frameworks [9, 10, 14, 15]. Additionally, results suggest that most, but not all, relevant revisions are detected during language translation, which suggests a potential benefit to pursuing cognitive testing following language translation. Although Lim and colleagues [23] identified the need for multiple revisions without language translation, we identified only one revision to achieve semantic and idiomatic equivalencies when language translation was not required.

There are several ways to interpret the differences in results. One possible explanation is that Lim and colleagues [23] pursued a transnational cultural adaptation without translation (i.e., from North America to Singapore), whereas this study focused on cultural adaptation without translation for use within the same country in which the instrument was originally developed. It is possible that a transnational context resulted in a greater number of revisions required to achieve semantic equivalence without translation. Alternatively, differences in results may be attributed to use of questionnaire versus caregiver interview for cognitive testing, as was used by Lim and colleagues [23]. Questionnaires afforded feasible data collection but may have limited opportunities to ask clarifying and probing questions. Thus, results of this study may underestimate the revisions required in order to achieve semantic and idiomatic equivalencies for the non-translated (English) version.

### Item and conceptual equivalencies

Caregivers proposed a greater number of revisions for item and conceptual equivalencies in the Spanish YC-PEM pilot version. Study results lend preliminary evidence that is contrary to the hypothesis that a similar number of revisions would be required for both versions.

#### Acculturation and language considerations

Group differences in the amount of feedback provided may suggest that fewer revisions are required to culturally adapt a measure without translation, particularly for use with a cultural group residing in the country in which the instrument was developed; however, this finding should be interpreted with caution due to the potential confounding effect of acculturation status. Skewed distributions for acculturation status in each group of English versus Spanish speakers may suggest that there is a potential effect of acculturation status on the number of revisions needed to achieve item and conceptual equivalencies. Thus, results may underestimate the impact of culture on cultural adaptations without language translation.

To our knowledge, no prior studies have examined the effect of acculturation status on the number of revisions required to achieve cultural equivalence of a measure. However, prior studies have examined the relation between acculturation and how caregivers conceptualize child development [36]. Study findings indicated that concepts of child development vary across Mexican-American acculturation levels as well as between highly acculturated Mexican-American caregivers and Anglo-American caregivers when controlling for socioeconomic status (SES) [36]. These findings suggest that Mexican culture may potentially influence caregiver perspectives about the concept of young children’s participation regardless of acculturation level, and thus revisions may be required across acculturation levels when culturally adapting measures. Additionally, concepts of child development vary across acculturation levels for high-SES participants, but not for low-SES participants [36]. Hence, future studies might sample across acculturation levels and control for income and education levels in order to further examine the influence of acculturation on revisions required to achieve cultural equivalence of measures, such as the YC-PEM. Employing online YC-PEM completion (e.g., via personal computer or iPad during service visits) followed by caregiver group interviews might increase feasibility and improve data quality.

Language may also influence cultural expression. For example, Arcia, Reyes-Blanes, and Vazquez-Montilla [37] found that participants placed emphasis on different Mexican cultural values depending on the particular interview language (i.e., Spanish or English). Niemann, Romero, Arredondo, and Rodriguez [38] suggested that language preference may be indicative of in-group or out-of-group discrimination based on language, and that as a result of discrimination, different cultural values may be emphasized. These findings suggest that cultural adaptations of instruments with and without translation will likely result in different types of revisions. Thus, future research should examine the impacts of language on cultural expression and the interaction between language and acculturation level on culturally adapting measures. Future studies might add a measure to capture discrimination, such as the Hispanic Stress Inventory [39], in order to examine this interaction effect.

#### Item equivalence

Feedback on the Spanish YC-PEM indicated that revisions necessary to achieve item equivalence primarily related to the addition or deletion of activity examples listed for each activity type. Given that the original YC-PEM activity types are fairly broad in nature, these categories may be deemed applicable across multiple cultural contexts due to their more generic nature. Common suggestions for revisions to activity examples pertained to self-care, educational activities, celebrations, and religious gatherings. These findings are consistent with identified Mexican cultural values pertaining to the values of responsibility, education, celebration, and familialism [37, 38, 40].

Additionally, participant feedback emphasized the social and emotional aspects of their child’s participation, which is consistent with established Mexican cultural values [37, 38, 40]. Because these features are not clearly captured in original YC-PEM activity types and examples, the emphasis on these qualities may warrant the addition of examples to further operationalize the dimension of involvement or the reframing of category descriptions in order to better capture these elements when assessing for a child’s participation in activities.

#### Conceptual equivalence

Conceptual equivalence concerns were identified among most participants, lending preliminary support for addressing conceptual equivalence regardless of language and acculturation level. Participants in this study commonly conceptualized “involvement” as requiring skills or some level of independence by the child. Hence, participants often indicated that items were not relevant based on the child’s young age or disability status. In a study by Arcia, Reyes-Blanes, and Montilla [37] that examined the impacts of disability on cultural values, caregivers of children with disabilities placed higher value on “being independent.” These findings contrast with Mexican cultural values for interdependence [37, 38, 40]. However, Arcia et al. [37] noted that caregivers used labels of “independence” to indicate internalization of caregiver values (e.g., respect, strong ties to caregivers), which contrasts with common definitions of independence pertaining to autonomous child behaviors. Therefore, the notion of “independence” may be more in line with caregiver values typically associated with Mexican culture including familialism (e.g., respect, strong ties to caregivers), work ethic, responsibility, and education [37, 38, 40]. Thus, findings from Arcia et al. [37] may be reflected in caregiver feedback from this study, which indicates that the concept of participation is associated with independence. Participant feedback further supports this through the emphasis placed on social relationships and emotional sharing, which aligns with values of familialism and responsibility.

Therefore, study findings and prior literature may indicate that framing participation more explicitly in terms of co-occupation is more in line with the conceptualization of participation in Mexican culture. Co-occupation involves shared engagement in daily activities resulting in shared meaning [41]. Although the YC-PEM implies co-occupation as young children typically participate in activities with a caregiver, this idea may need to be made more explicit throughout YC-PEM instructions and participation sections (e.g., by providing examples and reframing participation category descriptions).

### Feasibility of cultural adaptation

As noted in previous studies [9, 11], one of the greatest barriers to producing culturally adapted measures is the resource-intensive nature. Despite resource requirements, cultural adaptation of existing measures remains more time and cost effective than creating new measures specifically for the targeted culture [9, 11]. Thus, examining the feasibility of the processes used in this study can help inform decision making about how to pursue future cultural adaptation work in ways that minimize cost and maximize quality.

#### Language translation

Language translation is a costly phase. In this study, costs were mitigated by recruiting bilingual, but not professional, translators. Reduced costs ensured that resources were available to undertake the full process and make enhancements to improve rigor.

During language translation, we found that synthesis and committee review phases were the most time intensive; however, examination of the discrepancy rate among translators demonstrates that these processes serve to systematically reduce discrepancies, thus suggesting an increase in translation quality. High translation quality is further supported by Aim 1 results, which show low revision rates related to semantic and idiomatic equivalencies following the translation period. Thus, for cultural adaptations when language translation is required, the language translation phase is critical to ensuring semantic and idiomatic equivalencies.

We recruited three forward translators, which exceeds the minimum of two translators recommended by most established translation guidelines [9, 10, 14, 15]. Although the addition of a third translator increased baseline costs, it potentially reduced the time investment with respect to language translation. Specifically, less time was needed for synthesis and committee review processes, because principles of majority rules could be applied when issues of language preference arose. Therefore, priorities of time or cost may help inform decisions surrounding the numbers of translators used in future cultural adaptations with language translation.

#### Cognitive testing

The use of early intervention service providers as the primary recruitment method increased access to eligible families; however, recruitment and data collection time increased due to provider constraints (e.g., limited time within therapy sessions, cancelled visits) in comparison with direct participant interaction by research staff [42].

Additionally, most eligible families who declined participation cited being “too busy/stressed.” Given that all eligible caregivers had children with a disability or delay, this may have occurred due to caregiver burden associated with caring for a child with a disability or delay. Alternatively, data collection occurred in the winter season when caregiver demands are high due to holidays and illness. Thus, future research should consider expanding the targeted population to include children with and without disabilities and delays and/or sampling during different seasons in order to increase recruitment rates and ultimately feasibility.

Due to resource constraints, PDF and paper versions of study materials were issued in lieu of pursuing online data collection. Consequently, errors in YC-PEM completion (e.g., incomplete or incorrectly completed items) occurred in both PDF and paper formats and across all participant cases. These errors did not occur in prior studies when the YC-PEM was administered online, because the online versions include automated prompts to guide participant completion [28]. Thus, feasibility and data quality can be enhanced with online data collection (e.g., via personal computer or iPad during service visit). However, cognitive testing may be enhanced via interviews as the questionnaire format used in this study provided limited opportunities to pose clarifying or follow-up questions. Thus, to balance feasibility with data quality, future studies might employ online YC-PEM administration followed by group interviews to confirm findings.

### Study limitations

Study results are preliminary and should be applied with caution due to several study limitations. First, small sample size and homogeneity did not permit parametric testing to compare language subgroups according to acculturation status and number of required revisions to achieve cultural equivalence. Cognitive testing using questionnaire versus interview format limited the ability to reach saturation with respect to participant feedback. Additionally, although participants completed cognitive testing on the daycare/preschool section of the YC-PEM, none of the children reported on for this study were enrolled in a center-based daycare/preschool program at the time of study enrollment. Thus, participant feedback for this section may have been restricted due to limited exposure to the daycare/preschool setting. Finally, reliable data on precise cost estimates for this study were not accessible and thus limited specificity in the examination of feasibility. Thus, more robust cost-benefit analyses are recommended for future studies.

## Conclusions

Study findings provide preliminary evidence of the revisions required to achieve cultural equivalence for English and Spanish versions of the YC-PEM prior to use with caregivers of young CSHCN of Mexican descent. Findings suggest greater revisions are required to achieve cultural equivalence of a translated pediatric functional assessment, though there is need for researchers to address conceptual equivalence in cultural adaptations with and without language translation. This next phase is underway and will include online data collection for YC-PEM completion and caregiver interviews during cognitive testing to improve data quality and diversify study enrollment according to acculturation status and SES.

## Abbreviations

ARSMA-II: Acculturation Rating Scale for Mexican Americans-II
CSHCN: Children with Special Health Care Needs
EI: Early intervention
SES: Socioeconomic status
YC-PEM: Young Children’s Participation and Environment Measure
